# Efficacy and Safety of Catheter Ablation for Atrial Arrhythmias in Solid Organ Transplant Recipients: A Single‐Center Experience

**DOI:** 10.1002/clc.70345

**Published:** 2026-05-20

**Authors:** Piotr Gardziejczyk, Martyna Skrzyńska‐Kowalczyk, Marta Skowrońska, Ewa Wlazłowska‐Struzik, Pawel Szymkiewicz, Piotr Pruszczyk, Jakub Baran

**Affiliations:** ^1^ Division of Clinical Electrophysiology, Department of Internal Medicine and Cardiology Medical University of Warsaw Warsaw Poland

**Keywords:** atrial arrhythmia, catheter ablation, solid organ transplant

## Abstract

**Introduction:**

Atrial arrhythmias are common among solid organ transplant recipients and independently associated with increased morbidity, mortality, and impaired graft survival. Pharmacological management is substantially limited by interactions with immunosuppressive therapy. Despite growing clinical need, data on the feasibility, safety, and efficacy of catheter ablation in this population remain scarce.

**Methods:**

Single‐center, retrospective, matched cohort study. All consecutive solid organ transplant recipients who underwent catheter ablation of symptomatic left atrial arrhythmias between July 2021 and June 2024 were included. Each was matched 1:2 to non‐transplant controls by age, sex, BMI, arrhythmia type, and ablation modality. All procedures were performed using contemporary techniques: pulsed field ablation (PFA) or very high‐power short‐duration (vHPSD) radiofrequency ablation.

**Results:**

Sixty‐six patients were included: 22 transplant recipients and 44 matched controls. Arrhythmia‐free survival at 12 months after a 2‐month blanking period was 68.2% versus 76.2% (*p* = 0.49; HR 1.57, 95% CI 0.60–4.12). Periprocedural complications occurred in 9.1% versus 4.6% (*p* = 0.60); no cases of stroke, cardiac tamponade, or access site complications requiring surgical intervention were recorded in either group. In the kidney transplant subgroup (*n* = 17), eGFR remained stable at 12 months (41.8 ± 12.1 vs. 43.8 ± 15.3 mL/min/1.73 m², *p* = 0.34), with no clinically significant decline in renal function observed.

**Conclusion:**

Catheter ablation for complex atrial arrhythmias in solid organ transplant recipients was feasible using contemporary ablation techniques, without major procedural complications, and no excess safety or efficacy signal was observed at 12 months relative to matched controls. Prospective multicentre studies are needed to confirm these observations.

## Graphical abstract

Catheter ablation for complex atrial arrhythmias in solid organ transplant recipients is feasible using contemporary techniques, with arrhythmia‐free survival and periprocedural complication rates comparable to matched non‐transplant controls.

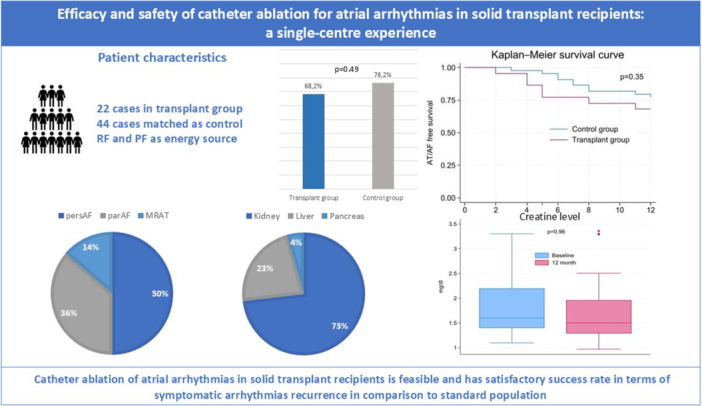

**Abbreviations**
AF: atrial fibrillation
AT: atrial tachycardia
ICE: intracardiac echocardiography
LA: left atrium
MRAT: macro reentrant atrial tachycardia
PAF: paroxysmal atrial fibrillation
PeAF: persistent atrial fibrillation
PFA: pulse field ablation
PVI: pulmonary vein isolation
RF: radiofrequency
vHPSD: very high power short duration

## 1 Introduction

Atrial arrhythmias, including atrial fibrillation (AF) and macro‐reentrant atrial tachycardia (MRAT), are common among solid organ transplant recipients and independently associated with increased morbidity, mortality, and impaired graft survival [1, 2, 3]. Their management is particularly challenging in this population: lifelong immunosuppressive therapy required to prevent graft rejection alters drug metabolism and increases the toxicity risk of antiarrhythmic agents, substantially narrowing pharmacological options [4]. Given the limited pharmacological alternatives, catheter ablation represents a particularly important therapeutic option in this population, yet it remains underutilized in clinical practice [5]. Data on the feasibility, safety, and efficacy of catheter ablation in this population remain scarce and limited to small single‐center series [6, 7]. No study has reported on the use of contemporary ablation technologies—pulsed field ablation (PFA) or very high‐power short‐duration (vHPSD) radiofrequency ablation—in this population. This uncertainty has direct clinical consequences, as it leaves physicians without evidence‐based guidance for a growing patient population.

Transplant recipients have been largely excluded from clinical trials evaluating catheter ablation for atrial arrhythmias. Key procedural concerns in this population include prolonged procedure times, fluid overload, iodine contrast‐related nephrotoxicity, bleeding and thrombotic risk, and the heightened susceptibility to infection inherent to immunocompromised patients [8, 9, 10].

Recent technological advances offer potential solutions to several of these transplant‐specific concerns. Cryoballoon ablation, while effective in the general population, usually requires iodine contrast administration — a relevant nephrotoxic risk in renal transplant recipients with already compromised graft function — although contrast‐free cryoballoon protocols have also been described [11]. Conventional RF ablation involves prolonged procedure times and significant intraprocedural fluid administration, both of particular concern in patients with reduced fluid tolerance and infection susceptibility. vHPSD RF ablation and PFA substantially reduce procedure times compared with conventional approaches [12, 13, 14], directly mitigating fluid overload risk. PFA offers an additional advantage through its cardioselective mechanism, which minimizes collateral tissue injury [15]. Together, these properties make contemporary ablation techniques potentially relevant to the specific procedural concerns encountered in solid organ transplant recipients.

The aim of this study was to characterize the safety and efficacy of catheter ablation for complex atrial arrhythmias in solid organ transplant recipients.

## 2 Methods

### 2.1 Study Design and Population

This was a single‐center, retrospective, observational matched cohort study. The case group consisted of all consecutive solid organ transplant recipients who underwent catheter ablation of symptomatic left atrial arrhythmias at our center between July 2021 and June 2024. Patients were identified through a review of the medical procedural database. No exclusion criteria were applied; all eligible patients identified during the study period were enrolled. No missing data were encountered, as procedural and follow‐up records were complete for all patients. As with all retrospective single‐center studies, the risk of selection bias cannot be entirely excluded and should be considered when interpreting these results.

Given the small sample size and the absence of prior data on ablation outcomes using contemporary techniques in this population, this study was designed as exploratory. No formal sample size calculation was performed; the analysis includes all eligible transplant recipients treated at our center during the study period.

All procedures involving human participants were conducted in accordance with the Declaration of Helsinki. Written informed consent was obtained from all participants. The study protocol was reviewed and approved by Ethics Committee of the Medical University of Warsaw (KB/19/2025). A waiver of additional informed consent for retrospective data analysis was granted, as data were collected as part of routine clinical care and analyzed in anonymised form.

### 2.2 Control Group Selection and Matching

Each transplant recipient was matched in a 1:2 ratio to non‐transplant patients who underwent catheter ablation at our center during the same study period. Matching was performed manually without replacement on the following variables: age (± 5 years), sex, body mass index (± 3 kg/m²), arrhythmia type, and ablation modality. Each control patient was assigned to only one transplant recipient. The 1:2 ratio was selected to maximize statistical power given the limited size of the transplant group. Matching was performed by an investigator blinded to clinical outcomes. When multiple eligible controls met the matching criteria for a given transplant recipient, the first two identified in chronological order of their procedure date were selected. Exact matching was enforced for arrhythmia type and ablation modality; age and BMI were matched within prespecified tolerance ranges (± 5 years and ± 3 kg/m², respectively). Post‐matching balance between groups was assessed by comparing baseline characteristics as presented in Table 1.

**Table 1:** Patients characteristics.

Baseline patient characteristics			
Parameter	Solid transplant recipients	Control	*p*
Patients, *n*	22	44	
Age, years, median (IQR)	65 (55; 69)	66.5 (60.5; 69.5)	0.57
Female, *n* (%)	7 (31.8)	12 (28.6)	0.79
BMI, kg/m^2^, mean (SD)	27 (3.8)	28.1(4)	0.31
Creatinine mg/dL, median(IQR)	1.6 (1.28; 2.2)	1.0 (0.9; 1.2)	< 0.01
LVEF, %, mean (SD)	56.1 (6.4)	57.7 (6.9)	0.32
LA diameter, mm, mean (SD)	45.2 (6.2)	41.4 (5.9)	0.02
LA volume, mm, mean (SD)	49.3 (11.9)	39.3 (11.2)	< 0.01
CHADS‐VA score, mean (SD)	3.4 (1.3)	2.2 (1.2)	< 0.01
Paroxysmal AF, *n* (%)	8 (36.4)	16 (36.4)	1
Persistent AF, *n* (%)	11 (50)	22 (50)	1
MRAT, *n* (%)	3 (13.6)	6 (13.6)	1
Congestive heart failure, *n* (%)	17 (77.3)	20 (45.5)	0.02
Hypertension, *n* (%)	15 (68.2)	34 (77.3)	0.25
Diabetes mellitus, *n* (%)	9 (40.9)	5 (11.3)	< 0.01

Abbreviations: BMI, body mass index; LA, left atrium; LVEF, left ventricular ejection fraction.

### 2.3 Periprocedural Management

Immunosuppressive therapy was continued without modification in all transplant recipients. All patients received intravenous hydration (1000 mL) on the day of the procedure. No routine antibiotic prophylaxis was administered. Patients treated with vitamin K antagonists underwent ablation on uninterrupted anticoagulation with a therapeutic INR; patients treated with NOACs withheld the morning dose on the day of the procedure, in accordance with institutional practice throughout the study period. Transoesophageal echocardiography was performed in all cases before the procedure to exclude left atrial thrombus. Periprocedural management did not differ between groups.

### 2.4 Vascular Access

All procedures were performed via three venous access lines placed in the right femoral vein under ultrasound guidance. No arterial access was used in any case.

### 2.5 Ablation Protocol

Heparin was administered after vascular access, prior to transseptal puncture, with further doses titrated to activated clotting time (ACT). Transseptal puncture was performed using a long SL0 sheath under fluoroscopic and/or intracardiac echocardiography (ICE) guidance. Additional left atrial lesion sets (anterior line, roof line, posterior wall isolation) and the use of a diagnostic mapping catheter were left to operator discretion. In cases of persistent AF with no restoration of sinus rhythm during ablation, electrical cardioversion was performed. After removal of left atrial sheaths, the pericardial space was assessed by ICE. A hemostatic figure‐of‐eight suture and pressure dressing were applied at the access site. Anticoagulation was resumed approximately 3–4 h after the procedure.

RF energy procedures were performed under analgosedation with fentanyl and midazolam using the CARTO electroanatomical mapping system. Pulmonary vein isolation was confirmed by the absence of pulmonary vein potentials and failure of atrial capture on pacing beyond the ablation line. All PFA procedures were performed under general anesthesia with ICE guidance. A minimum of four application pairs per pulmonary vein were delivered. Additional applications outside the pulmonary veins were performed under ICE guidance at operator discretion, primarily in cases of long‐standing persistent AF.

### 2.6 Outcomes

The primary outcome was freedom from any atrial tachycardia or atrial fibrillation (AT/AF) at 12 months after a 2‐month blanking period. Arrhythmia recurrence was defined as documentation of AT/AF on 12‐lead ECG or a minimum of 30 s of AT/AF on Holter monitoring. The secondary outcomes were assessed individually and included: stroke or transient ischemic attack (TIA), prolonged hospital stay (defined as hospital stay exceeding 1 day after the procedure), groin access complications requiring surgical intervention, pericardial effusion not causing tamponade, bleeding requiring transfusion, phrenic nerve injury, esophageal injury, pulmonary vein stenosis, readmission and significant renal function deterioration in the renal transplant subgroup, defined as a decrease in eGFR exceeding 25% from baseline. Individual components of the secondary endpoint are reported separately to aid interpretation. Renal outcomes were assessed exclusively in the renal transplant subgroup, as detailed in the statistical analysis section.

### 2.7 Follow‐up and Outcome Assessment

Follow‐up data were collected retrospectively from medical records for both groups. All patients were monitored at scheduled outpatient visits at 3, 6, and 12 months, with a 12‐lead ECG recorded at each visit and at least one 24‐h Holter monitor recording performed during the follow‐up period. Transplant recipients additionally attended transplant outpatient clinic visits, at which an ECG was performed when clinically indicated; any arrhythmia documentation from these visits was included in the analysis. Any externally documented ECG recording AT/AF in the context of symptoms was also considered for outcome ascertainment in both groups. Arrhythmia recurrence was defined solely on the basis of documented evidence; the absence of documented arrhythmia was considered as freedom from recurrence.

### 2.8 Statistical Analysis

Categorical variables are presented as counts and percentages and compared using the chi‐squared test or Fisher's exact test, as appropriate. Continuous variables are presented as mean ± standard deviation or median with interquartile range depending on distribution, assessed using the Shapiro‐Wilk test and visual inspection of histograms. Normally distributed variables were compared using the two‐tailed independent samples t‐test; non‐normally distributed variables were compared using the Mann‐Whitney U test. Time to first AT/AF recurrence was estimated using the Kaplan‐Meier method with log‐rank testing. Patients with no documented arrhythmia recurrence were censored at 12 months and classified as event‐free. All patients completed the prespecified 12‐month follow‐up period. Given the small sample size of the transplant group, multivariable adjustment was not performed. A p‐value of less than 0.05 was considered statistically significant.

## 3 Results

### 3.1 Patient Population

A total of 66 patients were included in the final analysis: 22 in the transplant group and 44 in the control group. Baseline characteristics are presented in Table 1. Groups were well matched for age (median 65 [IQR 55‐69] vs. 66.5 [IQR 60.5−69.5] years, *p* = 0.57), sex (31.8% vs. 28.6% female, *p* = 0.79), BMI (27.0 ± 3.8 vs. 28.1 ± 4.0 kg/m², *p* = 0.31), arrhythmia type, and ablation modality. The most common arrhythmia was persistent AF (50%), followed by paroxysmal AF (36.4%) and MRAT (13.6%), with identical distribution between groups.

Despite matching, several clinically relevant baseline differences were present between groups. The transplant group had significantly larger left atrial diameter (45.2 ± 6.2 vs. 41.4 ± 5.9 mm, *p* = 0.02) and left atrial volume (49.3 ± 11.9 vs. 39.3 ± 11.2 mL, *p* < 0.01), higher prevalence of congestive heart failure (77.3% vs. 45.5%, *p* = 0.02) and diabetes mellitus (40.9% vs. 11.3%, *p* < 0.01), and higher thromboembolic risk as assessed by CHA₂DS₂‐VA score (3.4 ± 1.3 vs. 2.2 ± 1.2, *p* < 0.01). Baseline serum creatinine was significantly higher in the transplant group (1.6 [IQR 1.28–2.2] vs. 1.0 [IQR 0.9–1.2] mg/dL, *p* < 0.01).

Transplant‐related characteristics are summarized in Table 2. Most transplant group patients had undergone kidney transplantation (*n* = 17, 77.3%), followed by liver (*n* = 5, 22.7%) and pancreas (*n* = 1, 4.5%). Most patients had received a single transplant procedure (*n* = 19, 86.4%); two patients had undergone two transplant procedures and one patient had undergone three. The most commonly used immunosuppressive agents were prednisone (86.4%), mycophenolate mofetil (81.8%), and tacrolimus (77.3%). No patient required modification of immunosuppressive therapy in relation to the ablation procedure. Prior to the procedure, 17 patients were on β‐blocker (77.3%), two patients were on amiodarone (9.1%) and one patient was on propafenone (4.5%) in the transplant group. In the control group 39 (88.7%) patients were on β‐blocker, 3 (6.9%) patients were on amiodarone and 4 (9.1%) patients were on propafenone. At 12‐month follow‐up, 15 patients were on β‐blocker (68.2%), five patients were on amiodarone (22.7%) and no patient was on propafenone in the transplant group. In the control group 39 (88.7%) patients were on β‐blocker, 3 (6.9%) patients were on amiodarone and 5 (11.4%) patients were on propafenone.

**Table 2:** Solid transplant related characteristics.

**Solid transplant**	
Kidney, *n* (%)	17 (77.3)
Liver, *n* (%)	5 (22.7)
Pancreas, *n* (%)	1 (4.6)
**Transplant procedures**	
1, *n* (%)	19 (86.4)
2, *n* (%)	2 (9.1)
3, *n* (%)	1 (4.6)
**Immunosupressive therapy**	
Prednisone, *n* (%)	19 (86.4)
Tacrolimus, *n* (%)	17 (77.3)
Mycophenolate mofetil, *n* (%)	18 (81.8)
Cyclosporin C, *n* (%)	4 (18.2)
Azathioprine, *n* (%)	1 (4.6)

### 3.2 Procedural Data

Procedural characteristics stratified by energy modality are presented in Table 3. RF ablation was performed in 15 transplant group patients (68.2%) and 30 control group patients (68.2%); PFA was performed in seven transplant group patients (31.8%) and 14 control group patients (31.8%). No statistically significant differences were observed between groups in procedure duration, fluoroscopy time, or fluoroscopy dose, regardless of energy modality. ICE was used in 81.8% of transplant group procedures and 95.5% of control group procedures (*p* = 0.07); ICE was used in all PFA procedures in both groups.

**Table 3:** Procedural characteristics (A) RF energy (B) PF energy (C) all group.

	Solid transplant recipients	Control	*p* value
**(A) Parameter**			
Number of procedures	15	30	
Procedure duration, min, median (IQR)	134 (115; 176)	110 (97; 135)	0.18
Fluoroscopy time, s	149 (100; 286)	74 (27; 222)	0.1
Fluoroscopy dose, mGy	6.94 (2; 18.7)	1.93 (1.1; 8.1)	0.1
ICE	11 (73)	28 (93)	0.07
**(B) Parameter**			
Number of procedures	7	14	
Procedure duration, min,	75 (65; 80)	60 (55; 75)	0.12
Fluoroscopy time, s	620 (552; 756)	455 (390; 651)	0.08
Fluoroscopy dose, mGy	13.3 (9.59; 18.5)	12.1 (7.25; 15.4)	0.5
Number of applications	50.4 (10)	46.2 (7.6)	0.28
Posterior wall isolation, n (%)	6 (85.7)	9 (60)	0.23
ICE	7 (100)	14 (100)	1
**(C) Parameter**			
Number of procedures	22	44	
Procedure duration, min, median (IQR)	117.5 (75; 150)	97 (75; 125)	0.2
Fluoroscopy time, s	249 (115; 552)	193 (46; 408)	0.16
Fluoroscopy dose, mGy	9.4 (3.9; 18.5)	5.63 (1.4; 12.5)	0.2
ICE, n (%)	18 (81.8)	42 (95.5)	0.07

Abbreviations: ICE, intracardiac echocardiography; PF, pulse field; RF, radiofrequency.

Pulmonary vein isolation was performed in all patients referred for AF ablation in both groups. Additional lesion sets beyond PVI were applied at operator discretion based on arrhythmia type and intraoperative findings.

In the transplant group, posterior wall isolation was performed in two paroxysmal AF patients (9.1%). Among persistent AF patients, posterior wall isolation was performed in 4 (18.2%) and an anterior line from the mitral valve to the right superior pulmonary vein in 3 (13.6%).

In the control group, posterior wall isolation was performed in three paroxysmal AF patients (6.8%). In persistent AF, posterior wall isolation was performed in nine patients (20.5%) and an anterior line from the mitral valve to the right superior pulmonary vein in one patient (2.3%).

The distribution of additional lesion sets did not differ significantly between groups (posterior wall isolation in paroxysmal AF: *p* = 1.00; posterior wall isolation in persistent AF: *p* = 1.00; anterior line in persistent AF: *p* = 0.1; any additional lesion set: *p* = 0.39).

### 3.3 Primary Outcome: Arrhythmia‐Free Survival

**Figure 1:**
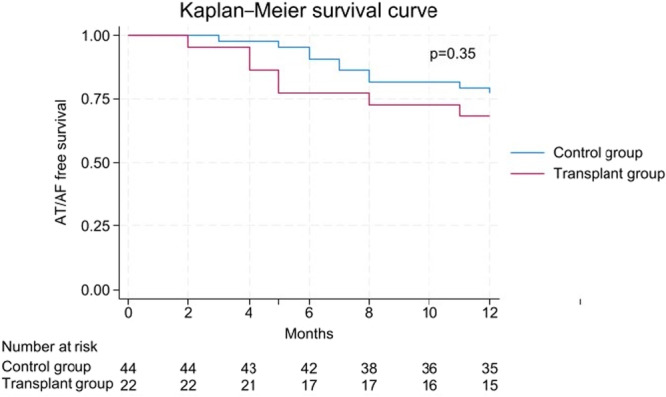
Kaplan–Meier survival curve. Kaplan–Meier curve comparing AT/AF free survival during 12 month follow up period. AT, atrial tachycardia; AF, atrial fibrillation.

**Figure 2:**
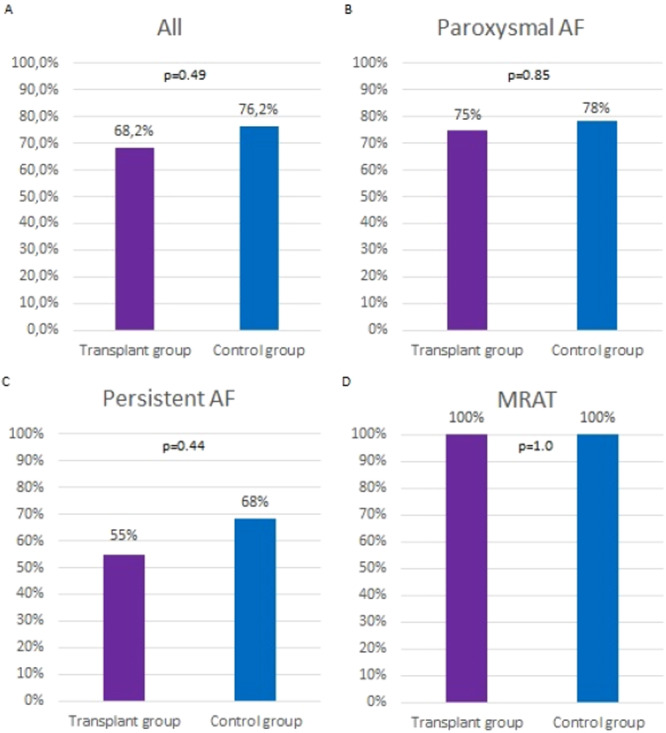
Catheter ablation efficacy in different arrhythmia types. (A) All arrhythmias combined, (B) paroxysmal AF, (C) persistent AF, and (D) MRAT. AF, atrial fibrillation, MRAT, macro‐reentrant atrial tachycardia.

Complete 12‐month follow‐up was obtained in all 66 patients. As illustrated in Figure 1, arrhythmia‐free survival at 12 months after the 2‐month blanking period was 68.2% in the transplant group and 76.2% in the control group (*p* = 0.49). The hazard ratio for arrhythmia recurrence in the transplant group relative to the control group was 1.57 (95% CI: 0.60–4.12). Figure 2 presents outcomes stratified by arrhythmia type: the highest success rates in both groups were observed in ablation of MRAT, followed by paroxysmal AF.

Secondary outcome: periprocedural complications

Periprocedural complications occurred in two of 22 patients (9.1%) in the transplant group and two of 44 patients (4.6%) in the control group (*p* = 0.6). No cases of cardiac tamponade, stroke, TIA, or groin access complications requiring surgical intervention were recorded in either group. No deaths occurred during the study period. No cases of pericardial effusion not causing tamponade, bleeding requiring transfusion, phrenic nerve injury, esophageal injury, pulmonary vein stenosis were observed in either group. No unplanned readmissions within 30 days occurred.

In the transplant group, one patient developed acute pulmonary oedema in the context of pre‐existing heart failure, procedural fluid administration, and restoration of sinus rhythm by electrical cardioversion, resulting in prolonged hospitalization. One patient developed pneumonia following ablation performed under general anesthesia, also resulting in prolonged hospitalization. In the control group, two patients experienced exacerbation of chronic heart failure requiring prolonged hospitalization, also in the setting of pre‐existing heart failure and electrical cardioversion.

Secondary outcome: renal function in the kidney transplant subgroup.

Renal function was assessed in the kidney transplant subgroup (*n* = 17). eGFR, calculated using the CKD‐EPI equation, was 41.8 ± 12.1 ml/min/1.73 m² at baseline and 43.8 ± 15.3 ml/min/1.73 m² at 12 months (*p* = 0.34). A clinically significant decline in renal function, defined as a decrease in eGFR exceeding 25% from baseline, was not observed in any of 17 patients.

**Figure 3:**
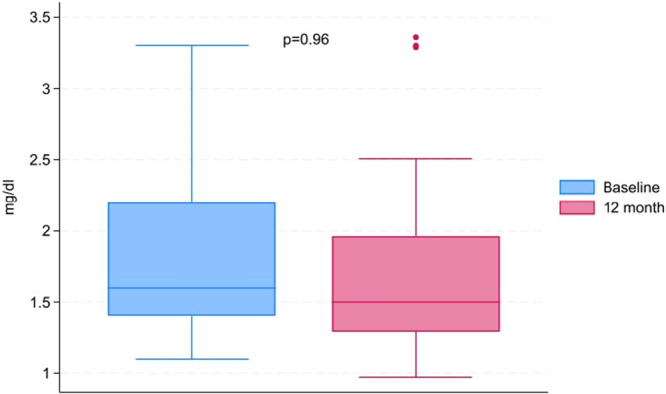
Differences in creatine level during 12 month follow up in the kidney transplant group.

Serum creatinine levels at baseline and at 12 months were comparable (1.6 [IQR 1.42–2.2] vs. 1.5 [IQR 1.3–2.0] mg/dL, *p* = 0.96), consistent with stable graft function (Figure 3). No patient required acute renal replacement therapy following the procedure.

## 4 Discussion

This study examined the outcomes of catheter ablation for complex atrial arrhythmias in solid organ transplant recipients compared with matched non‐transplant controls. The main finding was that catheter ablation was feasible in this population. Despite a less favorable baseline risk profile in the transplant group, observed outcomes did not differ extensively from those of matched controls. However, given the small sample size and wide confidence intervals, these findings should be interpreted as the absence of an overt excess safety or efficacy signal in this exploratory cohort, rather than evidence of equivalence.

### 4.1 Primary Outcome: Procedural and 12‐Month Success

Acute procedural success, defined as PVI and/or MRAT termination with documented bidirectional block, was achieved in all patients. At 12 months, arrhythmia‐free survival was 68.2% in the transplant group and 76.2% in the control group. The highest success rates in both groups were observed in ablation of left atrial MRAT, consistent with the ability to precisely identify and target the arrhythmia circuit in these cases.

Two prior studies have reported on arrhythmia‐free survival following catheter ablation in solid organ transplant recipients, both of which used conventional ablation technologies. Su et al. described outcomes in 14 kidney and hepatic transplant recipients undergoing radiofrequency ablation for atrial fibrillation, reporting 12‐month arrhythmia‐free survival of 71.4% and a periprocedural complication rate of 14.3% [7]. Keelani et al. reported 18‐month arrhythmia‐free survival of 69% in 16 kidney transplant recipients, with a complication rate of 6.3% [6]. The 12‐month arrhythmia‐free survival of 68.2% and complication rate of 9.1% observed in our cohort are broadly consistent with both prior series.

These similarities should be interpreted with important caveats. The marginal increase in sample size from 14 (Su et al.) to 22 patients in the current study represents a limited absolute gain in statistical power; neither our study nor the prior series is adequately powered to detect moderate differences in clinical outcomes. Accordingly, consistent numerical findings across studies reflect the overall feasibility of the procedure in this population rather than equivalence of outcomes between individual approaches. The current cohort is nonetheless the first to include contemporary ablation technologies—PFA and vHPSD RF ablation—which were not available at the time of Su et al. Both modalities offer substantially shorter procedure times compared with conventional point‐by‐point RF ablation, a property of particular relevance in transplant recipients given the procedural risks associated with prolonged fluid administration and general anesthesia. Notwithstanding these distinctions, the cumulative evidence from all three studies—encompassing 52 transplant recipients in total—converges on the same principal finding: catheter ablation is technically feasible and achieves acceptable short‐to‐medium‐term outcomes in carefully selected solid organ transplant recipients. The consistency of this finding across centers, time periods, and ablation technologies provides modest reassurance, while simultaneously underscoring the need for larger, prospective, multicentre studies to characterize the risk–benefit profile with appropriate statistical precision. Of note, a higher proportion of transplant recipients were receiving amiodarone at 12 months (22.7% vs. 6.9%), which may have pharmacologically suppressed recurrences that would otherwise have been detected, potentially inflating the arrhythmia‐free survival estimate in this group.

The transplant group in our study was heterogeneous, comprising predominantly kidney recipients, with a smaller number of liver and pancreas transplant recipients. Whether transplanted organ type independently influences ablation outcomes in this population remains uncertain. Kidney transplant recipients carry a specific burden of impaired renal function, CKD‐related cardiovascular remodeling, and exposure to calcineurin inhibitors, all of which may contribute to a more complex arrhythmia substrate and higher procedural risk. Liver transplant recipients may present distinct hemostatic challenges related to synthetic liver function and perioperative coagulopathy. The limited number of recipients of each transplant type in our cohort precludes any organ‐specific subgroup analysis; this question warrants dedicated investigation in future studies.

### 4.2 Secondary Outcomes: Safety

Solid organ transplant recipients, particularly those with chronic kidney disease, carry an elevated baseline risk of both thromboembolic and bleeding complications [16], and increased rates of vascular access‐related events have been reported in this population in prior studies [17, 18]. Against this background, the absence of stroke, TIA, cardiac tamponade, or access site complications in our cohort is clinically relevant.

Periprocedural complications occurred in 9.1% of transplant group patients and 4.6% of controls. In the transplant group, one patient experienced prolonged hospitalization due to acute pulmonary oedema in the context of pre‐existing chronic heart failure, procedural fluid administration, and restoration of sinus rhythm by electrical cardioversion; one patient developed pneumonia following ablation performed under general anesthesia. Comparable cases of heart failure exacerbation were observed in the control group. Complication rates reported in prior studies were 14.3% (Su et al.) and 6.3% (Keelani et al.), consistent with the rate observed in our cohort.

All venous access was obtained under ultrasound guidance, consistent with evidence supporting ultrasound‐guided vascular access as a means of reducing access‐related complications regardless of operator experience [19]. Intracardiac echocardiography was used in the majority of procedures to guide transseptal puncture and catheter manipulation, in line with evidence supporting its role in reducing complications related to left atrial access [20, 21].

Brain MRI was not performed routinely before or after the procedure; therefore, silent cerebral ischemic events cannot be excluded.

### 4.3 Secondary Outcomes: Kidney Function

Renal function in the kidney transplant subgroup did not change significantly following the procedure. This subgroup warrants particular attention given the inherent vulnerability of the transplanted kidney to periprocedural insults. Routine ICE‐guided transseptal puncture and pulmonary vein assessment allowed contrast avoidance in all cases. Whether this contributed to renal preservation cannot be determined from the available data. Keelani et al. similarly reported preserved graft function following ablation in kidney transplant recipients, with one case of graft dysfunction occurring in the context of a procedural complication. These observations are broadly consistent with prior studies suggesting that catheter ablation does not adversely affect long‐term renal function.

A proportion of patients in the renal transplant subgroup underwent PFA. Concerns regarding PFA‐associated hemolysis and acute kidney injury have been documented in the literature [22, 23]. No hemolytic complications or acute kidney injury were observed in our cohort; however, given the limited number of patients treated with PFA in this subgroup, no conclusions regarding the renal safety profile of PFA can be drawn. Dedicated prospective studies are needed to adequately characterize this risk in transplant recipients.

The role of PFA in solid organ transplant recipients warrants specific consideration. Beyond its established cardioselectivity and the procedural time advantages described above, PFA has recently been shown to be feasible and effective for the treatment of persistent and long‐standing persistent AF using extended ablation strategies targeting the posterior wall and electrogram‐guided substrate, with favorable outcomes and an acceptable safety profile even with an increased number of applications [24]. Whether the cardioselective mechanism of PFA and its applicability to complex substrates confer meaningful clinical advantages in transplant recipients cannot be fully determined from the present data. The cohort included multiple arrhythmia types, lesion sets, and energy sources, and no modality‐specific conclusions can be drawn. This study was not designed or powered to compare PFA versus RF ablation.

## 5 Study Limitations

This study has several limitations that should be considered when interpreting the results. First, the retrospective, single‐center, observational design is inherently subject to selection bias. Transplant recipients referred for catheter ablation may represent a healthier subset with fewer comorbidities than those who were not, which may limit the generalizability of the findings.

Second, the sample size is small. The transplant group was heterogeneous in terms of organ type, with very few recipients of certain transplant types (e.g., a single pancreas transplant recipient), precluding subgroup analyses. Wide confidence intervals reflect the uncertainty inherent to small observational studies.

Third, despite matching on key clinical variables, residual confounding cannot be excluded. Post‐match imbalances persisted in left atrial size, heart failure, diabetes, CHA₂DS₂‐VA score, and serum creatinine—all established determinants of recurrence and procedural risk. Multivariable adjustment was not performed given the limited event count, and these residual differences constrain the interpretability of the between‐group comparison.

Fourth, the choice of additional lesion sets beyond pulmonary vein isolation was left to operator discretion, and RF ablation procedures were performed under analgosedation while PFA procedures were performed under general anesthesia, reflecting standard institutional practice for each modality but introducing a degree of procedural heterogeneity.

Fifth, the follow‐up protocol relied on intermittent ECG and Holter monitoring without continuous rhythm surveillance, which may underestimate the true recurrence in both groups. However, monitoring intensity may have differed: transplant recipients had additional clinic contacts that could have increased asymptomatic detection. These limitations highlight the need for prospective, multicentre studies with standardized follow‐up protocols and larger cohorts to provide more definitive evidence on the outcomes of catheter ablation in solid organ transplant recipients.

## 6 Conclusions

Catheter ablation for complex atrial arrhythmias in solid organ transplant recipients was technically feasible using contemporary ablation techniques without major procedural complications, and no excess safety or efficacy signal was observed at twelve months relative to matched controls. These findings provide preliminary evidence that catheter ablation may represent a viable therapeutic option in carefully selected transplant recipients, and support the design of prospective multicentre studies to confirm these observations.

## Author Contributions

All authors contributed substantially to the conception, design, analysis, and interpretation of the data.

## Funding

The authors have nothing to report.

## Conflicts of Interest

The authors declare no conflicts of interest.

## Data Availability

The data that support the findings of this study are available from the corresponding author upon reasonable request.
